# Application of Nanometal Oxides In Situ in Nonwoven Polyester Fabric for the Removal of Bacterial Indicators of Pollution from Wastewater

**DOI:** 10.1155/2014/950348

**Published:** 2014-02-02

**Authors:** Sohair I. Abou-Elela, Hanan S. Ibrahim, Mohamed M. Kamel, Mohamed Gouda

**Affiliations:** ^1^Water Pollution Research Department, National Research Center, P.O. Box 12622, Cairo, Egypt; ^2^Chemistry Department, Faculty of Science, King Faisal University, Al-Hassa, Saudi Arabia; ^3^Textile Research Division, National Research Center, Cairo, Egypt

## Abstract

The objective of this study is to investigate and assess the use of in situ deposit nanosilver (nAg_2_O) or nanocopper oxides (nCuO) into nonwoven polyester fabric (NWPF) as a safe and effective antibacterial filter of pollution from domestic wastewater. The bactericidal effect of both nAg_2_O and nCuO was examined against Gram-negative bacteria (*Escherichia coli*, *Salmonella typhi*) and Gram-positive bacteria (*Enterococcus faecalis*, *Staphylococcus aureus*) using agar diffusion disk method. In addition, the capability of nAg_2_O and nCuO as disinfectants for secondary treated domestic wastewater was investigated as a case study. Transmission electron microscope (TEM) confirmed the formation of nAg_2_O and nCuO particles with average particle sizes of 15 and 41 nm, respectively. Disk diffusion results showed that nAg_2_O had a higher bactericidal effect than nCuO. Moreover, the disinfection of secondary treated wastewater using 1.27 mg/cm^3^ of nAg_2_O in the nonwoven fabric was capable of hindering 99.6% and 91.7% of total and fecal coliforms within 10 minutes with a residual value of 18 and 15 MPN-index/100 mL, respectively. The residual total and fecal coliform concentrations were far less than that stated in the national and international limits for wastewater reuse in agriculture purpose.

## 1. Introduction

Municipal wastewater is one of the major sources of aquatic pollution, particularly in developing countries. Microbial contamination of water poses a serious threat to public health. Accordingly, wastewater should be disinfected to prevent the transmission of infectious diseases and to ensure that water is safe for human contact and the environment [1]. Although disinfection methods currently used in water and wastewater treatment can effectively control microbial pathogens, research in the past few decades has revealed a dilemma between effective disinfection and formation of harmful disinfection byproducts (DBPs). Chemical disinfectants commonly used by the water industry such as free chlorine, chloramines, and ozone can react with various constituents in natural water to form DBPs, many of which are carcinogens. In some literature, 600 DBPs have even been reported [2, 3]. The resistance of some pathogens, such as *Cryptosporidium* and *Giardia*, to conventional chemical disinfectants requires an extremely high disinfectant dosage, leading to aggravated DBP formation. Therefore, there is an urgent need to reevaluate conventional disinfection methods and to consider innovative approaches that enhance the reliability and robustness of disinfection while avoiding DBP formation [4].

Moreover, the emergence of nanoscience and nanotechnology in the last decade presents opportunities for exploring the bactericidal effect of metal nanoparticles. This has been attributed to their small size and high surface to volume ratio, which allows them to interact closely with microbial membranes, and is not merely due to the release of metal ions in solution [5]. Many of the present problems concerning water quality could be resolved or greatly ameliorated using nanosorbents, nanocatalysts, bioactive nanoparticles, nanostructured catalytic membranes, and nanoparticles enhanced filtration along with other products and processes resulting from the development of nanotechnology. Innovations in the development of novel technologies to disinfect water are among the most exciting and promising approaches.

Furthermore, nanotechnology-derived products that reduce the concentrations of toxic compounds to sub-ppb levels can be used to support the achievement of water quality standards and health advisories [6]. Metal nanoparticles with bactericidal activity can be immobilized and coated on surfaces, which may find application in various fields, that is, medical instruments and devices, water treatment, and food processing. Metal nanoparticles may also be combined with polymers to form composites for better utilization of their antimicrobial activity [7–10].

The objective of this study was to in situ deposit nanometal oxides, such as nanoparticles of silver and copper oxides, into nonwoven polyester fabric, to investigate and asses the capability of nanoparticles, deposited in an innovative fabric, as disinfectants for the removal of bacterial indicators of pollution from secondary treated domestic wastewater.

## 2. Experimental Methods

### 2.1. Materials

Nonwoven polyester fabric (NWPF) was purchased from the local market. Silver nitrate and copper chloride were purchased from Merck (Germany). The used hydrogen peroxide (H_2_O_2_) and other laboratory chemicals were of analytical grade purchased from Sigma-Aldrich Chemical Company.

### 2.2. Methods

#### 2.2.1. In Situ Deposition of Nanosilver Oxide or Nanocopper Oxide into Nonwoven Polyester Fabric

The nanometal oxides deposition was performed by padding the polyester fabric samples in an aqueous solution containing 50 mmol of metal salts solution (silver nitrate or copper chloride) and 1.5 wt. % (w/v) of polyvinyl pyrrolidone (PVP) as stabilizing agent. The fabric was then squeezed to a wet pick up 100%. The fabric was padded twice in the reducing-oxidizing bath containing 4 g/L sodium hydroxide and 10 mL H_2_O_2_ (35%) at pH 9.5, then squeezed to a wet pick up of 100%. The treated fabric was dried at 50°C for 10 minutes, then thoroughly washed with water for 45 minutes at 50°C and dried for 20 minutes at 50°C.

#### 2.2.2. Characterization of Treated Polyester Fabric

Treated polyester fabric with nanometal oxides was examined qualitatively and quantitatively using energy dispersive X-ray spectrum (SEM-EDX), coupled with a scanning electron microscope (type JXA-840—electron probe micro-analyzer—JOEL) and a transmission electron microscope (TEM) that gave images of cross-section of treated fabric samples. Total silver concentrations were determined according to Standard Methods for the Examination of Water and Wastewater [11], using an atomic absorption spectrometer, Varian SpectrAA (220), with a graphite furnace accessory and equipped with deuterium arc background corrector.

#### 2.2.3. Water-Leaching Test

Triplicate samples of one gram of fabric were transferred to shaking bottles with 10 mL de-ionized water. The bottles were then sealed with Para film lids and secured, rotated end-over-end for 48 h at 30 rpm, and then filtered through a 0.45 *μ*m membrane filter. Leached silver was analyzed in the filtrate [11]. For each series of metal measurements, an absorption calibration curve was constructed. It was composed of a blank and three or more standards from Merck (Germany). Accuracy and precision of the silver measurement were confirmed using external standard reference material 1643e, from National Institute Standards and Technology (NIST).

#### 2.2.4. Bacterial Strains

Two Gram-negative bacterial strains (*Escherichia coli* ATCC 11229 and *Salmonella typhi* ATCC 13311) and two Gram-positive bacterial strains (*Enterococcus faecalis *ATCC 29212 and* Staphylococcus aureus *ATCC 6538) were used as reference strains for studying the bactericidal effect of nAg_2_O and nCuO. All bacterial strains were grown overnight (37°C for 24 h) in nutrient broth (Himedia, India). The nutrient agar (Himedia, India) composed of peptic digest of animal tissue (5.0 g), sodium chloride (5.0 g), beef extract (1.5 g), yeast extract (1.5 g), and agar (15.0 g/L).

#### 2.2.5. Determination of the Efficiency of Treated Polyester Fabric Using Disk Diffusion Test

A comparison between treated NWPF with nAg_2_O and nCuO using a disk of 6 mm diameter of fabric was carried out using agar diffusion disk test [12]. 0.1 mL of each overnight grown bacterial suspension (10^4^–10^5^ CFU/mL) was spread over the surface of nutrient agar Petri dishes (Himedia, India). Disks of both nAg_2_O and nCuO were then placed over the surfaces of petri dishes. The dishes were inversely incubated at 37°C for 24 h. The antibacterial effect was qualified based on the formation of inhibition zone around the disks.

#### 2.2.6. Effect of Contact Time for Bacterium-Nanoparticle Interaction

Three overnight grown bacterial suspensions of *Staphylococcus aureus*,* Escherichia coli*, and *Salmonella typhi* with initial counts 3.1 × 10^4^, 4.6 × 10^4^, and 6.4 × 10^3^ CFU/mL, respectively, were examined. Three 26 cm^2^ surface area slides was immersed into three tubes containing 100 mL of examined bacterial suspensions at different contact times (15 min to 24 h). After each contact time, the count of each bacterial suspension was determined using poured plate count method according to Standard Methods for the Examination of Water and Wastewater [11].

#### 2.2.7. Application of Polyester Fabric Loaded with Nanometal Oxides as a Disinfectant for a Secondary Treated Domestic Wastewater (Case Study)

Domestic wastewater effluent treated in a Packed bed upflow anaerobic sludge blanket (P-UASB) followed by Inclined Plate Settler (IPS) then Multistage Roughing Fine Sand Filtration (MSRFF) was used as a real source for testing the efficiency of antibacterial filter [13]. Physicochemical and bacterial indicators (total coliforms and fecal coliforms) of raw and treated wastewater were determined. Slides with a 26 cm^2^ surface area were immersed in 200 mL of treated wastewater (25 samples) for a contact time of 10 min. Total and fecal coliforms were examined using the MPN-method according to Standard Methods for the Examination of Water and Wastewater [11].

## 3. Result and Discussion

### 3.1. Characterization of Treated Polyester Fabrics

**Figure 1:**
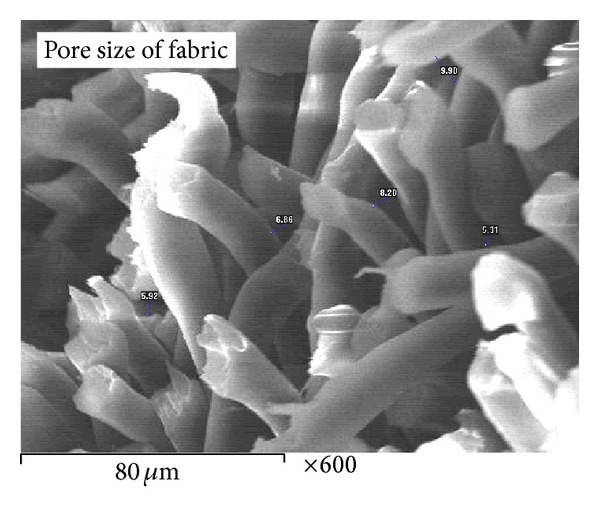
SEM of polyester fabric.

In situ deposition of nanometal oxides into NWPF with a thickness of 1.82 mm was used for disinfection of various microbial strains of Gram-positive and Gram-negative bacteria. Also, disinfection of a real secondary treated wastewater, as a case study, was investigated. Cross-section image of dried fabric was taken with a scanning electron microscope in high vacuum mode after coating with approximately 10 nm of gold to observe fabric asymmetry and its pore structure (Figure 1). It was found that the pore size of the fabric ranged from 5.3 to 9.9 *μ*m with an average pore size of 7.2 *μ*m.

**Figure 2:**
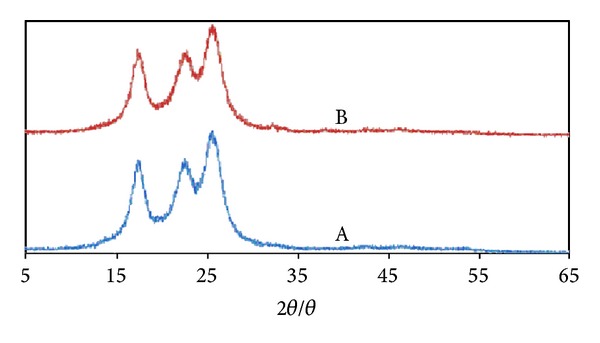
XRD patterns of polyester fabric (A) before loading and (B) after loading with nanometal oxides.

**Figure 3:**
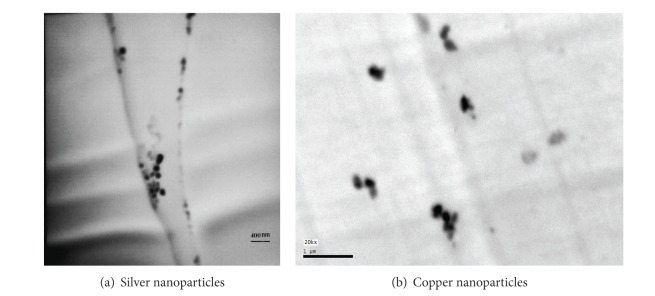
Transmission electron micrograph of cross-section fabric (a) after loading with nanosilver oxide and (b) after loading with nanocopper oxide.

In addition, the X-ray diffraction(XRD) patterns of the NWPF before and after loading with nanometal oxides are shown in Figure 2. The recorded XRD patterns indicated no change after loading with nanometal oxides and no relevant peaks of metals were observed. This indicated that metals did not affect fabric crystallinity. However, there was a slight shift in peaks position, which revealed that metals particles were incorporated in the fabric. TEM micrographs of fabric loaded with nanometal oxides (Figures 3(a) and 3(b)) confirmed the formation of nanosilver and nanocopper particles of about 15 nm and 41 nm on average. Furthermore, the total concentration of silver and copper in the treated fabric was 4635 and 4610 mg/kg, respectively.

### 3.2. Effect of the Two Types of Nanometal Oxides on the Removal of Bacteria Using Disk Diffusion Method

The two types of in situ nanometal oxides fabrics, namely, nAg_2_O and nCuO were examined for the removal of bacterial indicators of pollution using the disk diffusion test.  Table 1 shows that nAg_2_O had a higher bactericidal effect than nCuO against both Gram-negative and Gram-positive examined strains. It was also clear that both of nAg_2_O and nCuO have higher bactericidal effect against Gram-negative strains than Gram-positive strains. This might be due to the fact that Gram-positive bacteria are more resistant than Gram-negative bacteria since they have thicker cell wall. Our results are in good agreement with other studies [14, 15].

### 3.3. Mobility of Nano-Ag-Oxide and Its Effect on the Morphology of Polyester Fabric

It is recognized that nanoparticles may have undesirable and unforeseen effects on the environment and in the ecosystem [16, 17]. Therefore, water-leaching test was carried out in this study in order to estimate the actual mobility of nano-Ag-oxide that can be obtained by applying simple one-stage leaching test. The test involved mixing of the fabric with deionized water at a liquid to solid ratio (L : S) 10 : 1 and agitation of the mixture for 12, 24, and 48 h then filtration and determination of dissolved species in the filtrate. The results obtained showed that the concentration of silver in the leachate did not exceed 0.01 mg/L even after 48 h exposure. These results suggest that the bactericidal action requires a close contact of microorganisms with the microbial fabric filter rather than being due to the release of metal ions in solution [4, 5].

#### 3.3.1. Morphology of Fabric

**Figure 4:**
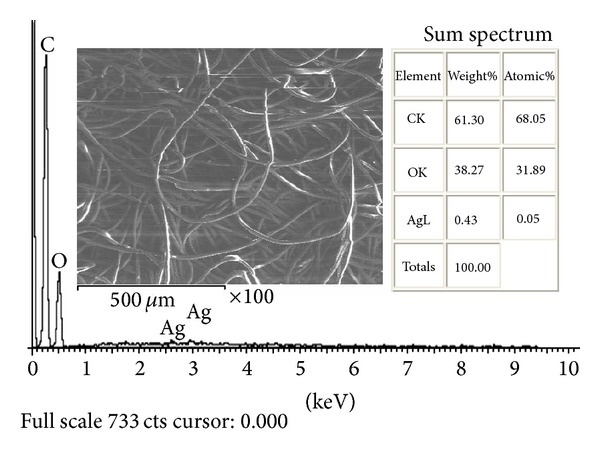
SEM-EDX analysis of fabric after nAg_2_O loading.

The morphology of fabric using the SEM micrographs after nano-Ag-oxide (nAg_2_O) loading is presented in Figure 4. The SEM image after loading with nAg_2_O indicated that it was well distributed inside the fabric. For the determination of silver content, energy-dispersive X-ray spectroscopy (EDX) analysis was conducted on 30 *μ*m of fabric. The calculated silver ratio was 0.43% which was almost in agreement with that determined by atomic absorption spectrometry (0.46%). This indicated that fabricated filter contains 1.27 mg/cm^3^ of silver nanoparticles.

**Figure 5:**
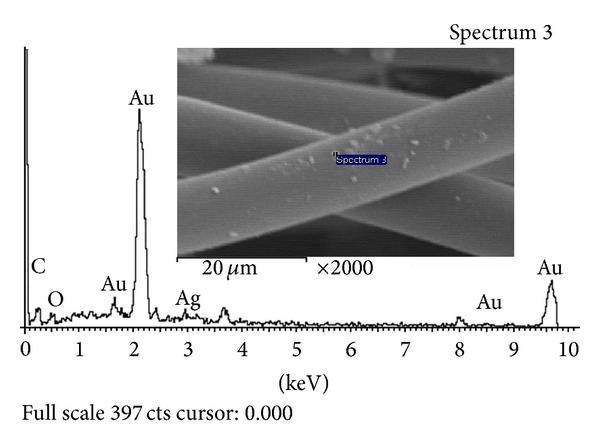
SEM-EDX micrograph with Au layer for fabric after nAg_2_O loading.

To confirm the morphology of the fabric used, it was treated with gold (Au) layer and recognized by Scanning Electron Microscopy (SEM). The energy dispersive spectrometer (EDS) spectrum for the fabric after firing was also recorded. The SEM image indicated that Ag was well distributed inside the fabric as shown in Figure 5.

### 3.4. Effect of Contact Time for Bacterium-Nano-Ag_2_O Interaction

The effect of contact time of nano-Ag_2_O fabric and three bacterial strains (*Staphylococcus aureus*, *Escherichia coli* and* Salmonella typhi*) at different contact times varying from 15 min to 24 h is shown in Table 2. A control of fabric without nano-Ag_2_O was used. The percent reductions in bacterial density after contact time of 60 min were 64.5, 73.9, and 67.2% for *Staphylococcus aureus*, *Escherichia coli*, and *Salmonella typhi*, respectively. Gradual increase of the contact time improved the removal efficiency of the different bacterial strains under investigation. An almost quantitative reduction of bacterial density was achieved after 180 min. The results indicated that n-Ag_2_O had a strong disinfectant effect on the examined bacterial strains. These results are in agreement with other studies [18, 19].

### 3.5. Efficiency of Nanosilver Oxide Fabric for Disinfection of Secondary Treated Domestic Wastewater: A Case Study

Physicochemical and bacterial indicators analyses of raw and secondary treated domestic wastewater effluent are shown in Table 3. Effluent from the integrated treatment system was disinfected using the NWPF loaded with n-Ag_2_O.

The effect of exposure time of nano-Ag-oxide incorporated in nonwoven polyester fabric, on the bacteria, was investigated. The results depicted in Table 4 show that the total coliform was reduced by around two orders of magnitude (from 5700 to 18 MPN-index/100 mL) after 10 min with a removal rate of 99.6%.

Also, fecal coliforms were reduced by one order of magnitude (from 260 to 15 MPN-index/100 mL) after 10 min. These residual values are far less than the concentration of coliforms stated in the Egyptian Code of Standards (501-2005) for reuse of wastewater for agricultural purposes (1000 MPN-index/100 mL) [20]. However, an exposure time of 30 minutes achieved almost complete removal for both total coliforms and fecal coliform. From this study, it can be concluded that a very satisfactory disinfection technology for a secondary treated wastewater can be achieved using nAg_2_O fabric filter with a suitable contact time. The results are in agreement with the studies for the use of silver as a disinfectant of generated wastewater especially from hospitals containing infectious microorganisms [21–24].

**Figure 6:**
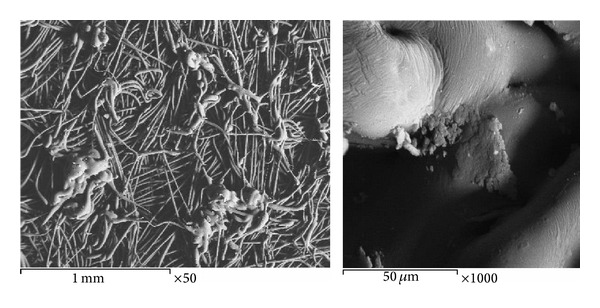
Cross-sections of treated polyester fabric taken after disinfection.

The results were also confirmed by the image shown in Figure 1 taken by a scanning electron microscope, where the image of the fabric filter has a small pore size (7.2 *μ*m), allowing it to act as a bacterial filter which reserves coliforms cells. Also, the small pore size of the filter allows more contact time between coliforms and nanoparticles that lead to more death of coliforms cells and consequently lead to nanoparticle incorporating in the cell membrane of microbes causing leakage of intracellular substances and eventually causing cell death [25–27].  Figure 6 shows the cross-section of treated polyester fabric taken after disinfection of coliforms where the dead cells were incorporated in the fabric and at the surface too.

## 4. Conclusion

The results indicated that bactericidal effect of nano-Ag-oxide is a promising alternative technique to the traditional chemical disinfectants which generate harmful disinfection byproducts. Silver nanoparticles are stable and are not washed away by water leaching test after 48 h. In situ deposition of nanometal oxides into nonwoven polyester fabrics proved to be a very effective antibacterial filter against *Escherichia coli*, *Salmonella typhi*, *Staphylococcus aureus*, and *Enterococcus faecalis *providing enough time to penetrate the multistage cell wall. However, after only 10 minutes and by using 1.27 mg/cm^3^ of nano-Ag-oxide fabric, more than 90% of coliforms removal was achieved from secondary treated wastewater. The residual value did not exceed 18 and 15 MPN-index/100 mL, respectively. These residual values are far less than 1000 MPN-index/100 mL which is the concentration of coliforms stated in the Egyptian Code of Standards (501-2005) for reuse of wastewater in agricultural purposes.

## Figures and Tables

**Table 1:** Effect of nAg_2_O and nCuO on microbial removal using agar diffusion disk test.

Types of polyester nanoparticles filters	Gram-negative		Gram-positive	
	*Escherichia coli *	*Salmonella typhi *	*Staphylococcus aureus *	*Enterococcus faecalis *
nAg_2_O	+++	+++	++	+
nCuO	++	+	++	−

+: low effect (<5 mm diameter); ++: moderate effect (5–15 mm diameter); +++: high effect (>15 mm diameter); −: no effect.

**Table 2:** Effect of contact time for bacterial reduction using nano-Ag-oxide.

Bacterial strain (CFU/100 mL)	Contact time	CFU/100 mL		% of removal
		Control of fabric without nAg_2_O	Fabric with nAg_2_O	
*Staphylococcus aureus *3.1 × 10^4^	*C_0_	3.0 × 10^4^	2.4 × 10^4^	**22.7**
	15 min	3.3 × 10^4^	2.1 × 10^4^	**32.3**
	30 min	3.4 × 10^4^	1.6 × 10^4^	**48.4**
	60 min	3.7 × 10^4^	1.1 × 10^4^	**64.5**
	120 min	4.1 × 10^4^	1.1 × 10^3^	**96.5**
	180 min	4.7 × 10^4^	8.6 × 10^2^	**97.2**
	24 h	5.1 × 10^4^	1.2 × 10^2^	**99.6**

*Escherichia coli *4.6 × 10^4^	*C*_0_	4.6 × 10^4^	3.2 × 10^4^	**30.4**
	15 min	4.8 × 10^4^	2.1 × 10^4^	**54.3**
	30 min	5.0 × 10^4^	1.6 × 10^4^	**65.2**
	60 min	5.4 × 10^4^	1.2 × 10^4^	**73.9**
	120 min	5.8 × 10^4^	4.2 × 10^3^	**90.8**
	180 min	6.1 × 10^4^	6.2 × 10^2^	**98.7**
	24 h	6.9 × 10^4^	1.1 × 10^2^	**99.8**

*Salmonella typhi *6.4 × 10^3^	*C*_0_	6.4 × 10^3^	5.2 × 10^3^	**18.8**
	15 min	6.5 × 10^3^	3.9 × 10^3^	**39.1**
	30 min	6.8 × 10^3^	3.7 × 10^3^	**42.2**
	60 min	7.1 × 10^3^	2.1 × 10^3^	**67.2**
	120 min	7.4 × 10^3^	1.0 × 10^3^	**84.4**
	180 min	7.7 × 10^3^	6.2 × 10^2^	**90.3**
	24 h	1.1 × 10^4^	10.1 × 10	**99.8**

*C_0_: the concentration at zero retention time; minutes: min; hours: h.

**Table 3:** Physicochemical and bacterial indicators of raw and treated wastewater*.

Parameters	Raw wastewater	Treated effluent**
pH	7.0 ± 0.14	7.3 ± 0.22

Total suspended solids (TSS) mg/L	200 ± 50.88	10.90 ± 4.39

Chemical oxygen demand (COD) mgO_2_/L	320 ± 52.9	60.30 ± 9.10

Biological oxygen demand (BOD) mgO_2_/L	190 ± 28.39	34.60 ± 5.23

Total coliform (MPN-index/100 mL)	2.8 × 10^7^	1.1 × 10^3^

Fecal coliform (MPN-index/100 mL)	1.5 × 10^7^	2.8 × 10^2^

*Average of 25 samples; **effluent from the integrated treatment system.

**Table 4:** Effect of contact time for reduction of total and fecal coliforms from secondary treated effluent using nano-Ag-oxide.

Time	Control		Nano-Ag-oxide			
	Total coliforms (MPN-index/100 mL)	Fecal coliforms (MPN-index/100 mL)	Total coliforms		Fecal coliforms	
			Conc. (MPN-index/100 mL)	% of removal	Conc. (MPN-index/100 mL)	% of removal
Zero time	4.1 × 10^3^	1.8 × 10^2^	4.1 × 10^3^	**0.0**	1.8 × 10^2^	**0.0**
2 min	4.6 × 10^3^	1.7 × 10^2^	1.1 × 10^3^	**73.2**	1.4 × 10^2^	**22.2**
5 min	5.1 × 10^3^	2.1 × 10^2^	7.5 × 10^2^	**81.7**	1.1 × 10^2^	**38.9**
10 min	5.7 × 10^3^	2.6 × 10^2^	1.8 × 10	**99.6**	1.5 × 10	**91.7**
15 min	6.2 × 10^3^	3.1 × 10^2^	1.5 × 10	**99.6**	1.0 × 10	**94.4**
20 min	6.4 × 10^3^	4.1 × 10^2^	1.1 × 10	**99.7**	5.3	**97.1**
30 min	6.8 × 10^3^	5.8 × 10^2^	1.0 × 10	**99.8**	5	**97.2**
